# Molecular Mechanisms Regulating Epithelial Mesenchymal Transition (EMT) to Promote Cancer Progression

**DOI:** 10.3390/ijms26094364

**Published:** 2025-05-03

**Authors:** Saima Ghafoor, Elizabeth Garcia, Daniel J. Jay, Sujata Persad

**Affiliations:** Department of Pediatrics, Faculty of Medicine and Dentistry, 3020R Katz Group Centre for Pharmacy and Health Research, University of Alberta, Edmonton, AB T6G 2E1, Canada; snotappl@ualberta.ca (S.G.); megarcia@ualberta.ca (E.G.); djaygarc@ualberta.ca (D.J.J.)

**Keywords:** EMT, intracellular signaling pathways, transcription factors, cancer progression, metastasis

## Abstract

The process of epithelial–mesenchymal transition (EMT) is crucial in various physiological/pathological circumstances such as development, wound healing, stem cell behavior, and cancer progression. It involves the conversion of epithelial cells into a mesenchymal phenotype, which causes the cells to become highly motile. This reprogramming is initiated and controlled by various signaling pathways and governed by several key transcription factors, including Snail 1, Snail 2 (Slug), TWIST 1, TWIST2, ZEB1, ZEB2, PRRX1, GOOSECOID, E47, FOXC2, SOX4, SOX9, HAND1, and HAND2. The intracellular signaling pathways are activated/inactivated by signals received from the extracellular environment and the transcription factors are carefully regulated at the transcriptional, translational, and post-translational levels to maintain tight regulatory control of EMT. One of the most important pathways involved in this process is the transforming growth factor-β (TGFβ) family signaling pathway. This review will discuss the role of EMT in promoting epithelial cancer progression and the convergence/interplay of multiple signaling pathways and transcription factors that regulate this phenomenon.

## 1. Overview of EMT

**Figure 1:**
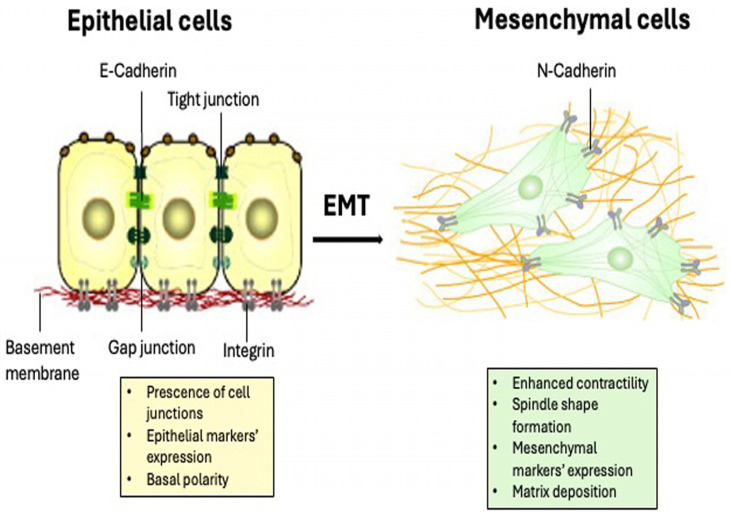
Epithelial to mesenchymal transition (EMT) and mesenchymal to epithelial transition (MET).

In the early 1980s, Elizabeth Hay first presented the idea of epithelial cells acquiring a mesenchymal phenotype while working on chick embryos. The process was primarily known as epithelial-to-mesenchymal transformation, which is now known as epithelial–mesenchymal transition (EMT) [1]. EMT is a reversible cellular event that transforms epithelial cells into a mesenchymal state. In this process, the epithelial cells in monolayer cultures gradually lose their shape to adopt a mesenchymal morphology. Moreover, mesenchymal cells can revert to an epithelial state in a reverse process known as mesenchymal–epithelial transition (MET) (Figure 1) [1,2]. EMT is reported to play crucial roles in several stages of embryogenesis including gastrulation, morphogenesis of tissues during development, and wound healing. EMT facilitates the formation of organs during development and the regeneration of tissue following injury. EMT enables epithelial cells to change into mesodermal or ectodermal cells during development of the embryo, aiding in the development of several organs like the heart, kidneys, and muscles. Complex organ architectures and appropriate tissue organization are made possible by this cellular plasticity [3,4]. In addition, activation of EMT in neoplastic cells leads to malignant progression of epithelial cancers (carcinomas). As the tumor progresses, this pleiotropic program endows individual cancer cells with various properties associated with malignancy [2,5]. EMT and MET, in all these processes, induce several fundamental alterations in the physiology and morphology of the cells. For instance, during the process of wound healing, the epithelial cells go through EMT to be motile and move as a group of cells for the reconstruction of the epithelial cell layer. By the end of the process, the activated EMT creates the quasi-mesenchymal cells which are reverted to their original phenotype (epithelial) by MET for re-establishing the integrity of the epithelial sheet [3,4]. Notably, the EMT program can be activated in a context-dependent manner and to various extents in epithelial cells under normal physiological conditions and in the progression neoplastic epithelial cells [3].

## 2. Types of EMT

**Figure 2:**
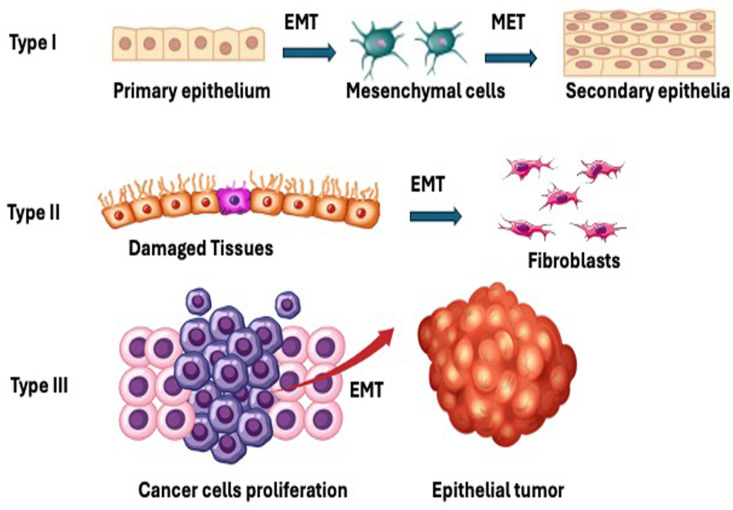
Types of EMT. Type I is associated with embryonic development, where the primary epithelium goes through EMT giving rise to primary mesenchymal cells that further goes through MET forming secondary epithelia. Type II is linked with inflammation and healing of wounds. However, if the primary inflammation is not recovered, it leads to fibrosis. Type III is associated with tumor progression of neoplastic cells.

Based on functional outcomes, EMT is classified into three types (Figure 2) [6].

### 2.1. EMT Type I

Physiological processes that include fertilization, embryonic development, and organ formation are associated with type I EMT. Type I EMT gives rise to mesoderm, endoderm, and neural crest cells [6,7,8]. EMT type I is an important process in embryogenesis that enables epithelial cells to become migratory mesenchymal cells, whereby they lose their polarity and adherence properties. As cells move across the primitive streak during gastrulation, this transformation creates the mesoderm. The ectoderm is formed by those cells that do not go through EMT, leading to the formation of nervous system and skin cells. Furthermore, at the neural plate boundary, EMT type I starts the development of neural crest cells, which allow them to move and delaminate before differentiating into a variety of tissues such craniofacial cartilage and peripheral neurons [8,9]. 

### 2.2. EMT Type II

EMT type II results in the formation of primitive mesenchymal cells that potentially undergo MET to form secondary epithelial cells that may further differentiate to various EMT-generating cell lineages such as astrocytes, adipocytes, chondrocytes, and osteoblasts. These cell types contribute to indirectly support EMT-related processes, especially through their involvement in fibrosis, inflammation, tissue remodeling, and cancer microenvironments. Type II EMT is associated with tissue repair, whereby the process generates myofibroblasts from epithelia to repair damaged tissues; if the injury is substantial and acute, the healing process is referred to as reparative fibrosis. In persistent chronic inflammation, aberrant myofibroblast production results in a gradual fibrosis that causes excessive ECM deposition, resulting in the destruction of organ parenchyma [6,7,8].

### 2.3. EMT Type III

EMT type III is associated with formation of fibroblast to regenerate new tissues damaged by injury, inflammation, or trauma. However, any changes in EMT type III may cause fibrosis and organ damage. Type III commonly occurs in neoplastic cells that lead to increased cell proliferation/tumor growth and tumor progression, with increased invasive and metastatic phenotypes [6,7,8].

## 3. Transcription Factors That Induce EMT and Regulate Invasion and Metastasis

During EMT, pivotal events include the loss of adherens junctions and downregulation of epithelial markers like cytokeratins and E-cadherin, accompanied by increased expression of mesenchymal markers such as fibronectin, N-cadherin, and vimentin. This transition results in the acquisition of a fibroblastic invasive phenotype and resistance to anoikis/apoptosis. See Table 1 for a summary of EMT markers [10].

The EMT process is tightly regulated by several transcription factors including Snail family transcriptional repressor 1 (snail 1) and Snail family transcriptional repressor 2 (Snail 2/(Slug), twist family BHLH transcription factor 1 (TWIST 1), twist family BHLH transcription factor 2 (TWIST2), Zinc finger E-Box binding homeobox 1 (ZEB1), Zinc finger E-Box binding homeobox 2 (ZEB2), paired related homeobox 1 (PRRX1), GOOSECOID (GSC), helix loop transcription factor (E47), Forkhead X box protein P2 (FOXC2), SRY box transcription factor 4 (SOX4), SRY box transcription factor 9 (SOX9), heart and neural crest derivatives expressed 1 (HAND1), and heart and neural crest derivatives expressed 2 (HAND2). In addition to these factors, EMT can be directly induced by a T-cell transcriptional factor (TCF) family member known as lymphoid enhancer binding factor 1(LEF1).

Of the above mentioned transcription factors, Snail family transcriptional repressor 2 (snail2), Zinc finger E-Box binding homeobox 1 (ZEB1), and twist family BHLH transcription factor 1 (twist) bind to the promoter regions of genes that are associated with cell–cell adhesion and cause the repression of their transcription. This comprises one of the early and main effects of EMT: loss of cell–cell adhesion [8,11,12]. These transcription factors also induce the upregulation of mesenchymal proteins, e.g., vimentin and N-cadherin, while downregulating epithelial proteins like E-cadherin. E-cadherin, a prominent cell adhesion protein in epithelial tissue, is highly implicated as it is frequently lost in carcinoma (epithelial tumor) progression, resulting in the tumor cells being able to detach from the parent tumor and become more migratory and invasive, an important step of cancer metastasis [11,13].

Transcriptional repressors, such as Snail family repressors, play an important role in EMT as they bind to promoter region of genes that encodes for E-Cadherin and represses the transcription of the protein [14]. Additionally, Snail family transcriptional repressor 1 (Snail 1) accumulation in the nucleus is highly associated with reduction in E-cadherin and the formation of metastatic properties in breast cancer [11,13,15]. During metastasis, Snail family transcriptional repressor 2 (Slug) was shown to play a vital role in EMT induction by suppressing the epithelial markers, e.g., E-cadherin, and promoting the mesenchymal markers like vimentin and N-cadherin, leading to a motile and invasive phenotype of the cells. Slug also promotes EMT during gastrulation and causes neural crest cell migration and formation. Slug facilitates the cellular mobility and plasticity required for these vital developmental processes by suppressing epithelial markers and enhancing mesenchymal traits [16,17]. When Snail 1 and Slug are overexpressed, they cause the induction of EMT, supporting tumor progression and metastasis [14,18].

Several layers of control are involved in the regulation of these important transcription factors. Numerous EMT-inducing transcription factors interact with each other in common pathways. However, studies have reported that inhibition of a single transcription factor is enough for blocking EMT [11,19]. In addition, overexpression of transcription factors that induces carcinogenesis or promotes progression is reported in many types of human cancers and their role/contribution in malignancy, including invasiveness and metastasis, has been well described (Table 2) [20,21].

## 4. The Canonical Wnt-β-Catenin Signaling Pathway

The canonical Wnt β-catenin signaling pathway plays an important role in embryogenesis including in cell proliferation, differentiation, and migration. This pathway is also crucial for cell apoptosis and the polarity of cells. Additionally, canonical Wnt signaling is important in driving the self-renewal process of stem cells of cancer and promoting tumor EMT that leads to metastasis and the progression of cancer [35].

The canonical Wnt signaling is activated when Wnt proteins bind to their cognate frizzled receptors in a complex with low-density lipoprotein receptor-related protein (LRPs) receptors. This results in the transduction of Wnt signals cross the plasma membrane, leading to initiation and entry into the cytoplasmic space. When Wnt ligands are not activated, β-catenin that is de novo synthesized in the cytoplasmic compartment and is phosphorylated by glycogen kinase-3β (GSK3β). GSK3β exists in complex with adenomatosis polyposis coli (APC) and Axin and phosphorylates β-catenin at its N-terminal domain at Ser 33, Ser 37, Thr 41, and Ser 45. Phosphorylation of β-catenin causes it to be ubiquinated and targeted for proteasomal degradation. Mechanistically, when Wnt binds its cognate receptors (Frizzled & LRP6), the protein Dishevelled (DVL) recruits the Axin/APC/GSK3β complex to the plasma membrane and causes the dismantling of the complex. This effectively prevents the phosphorylation and degradation of β-catenin by GSK3β, resulting in increased cytosolic levels of β-catenin and its translocation to the nucleus [11,36]. In the nucleus, the β-catenin binds to the transcription factor TCF which promotes the transcription of many proteins that include EMT-promoting or EMT-associated components, such as Snail1, Slug, and vimentin, resulting in the generation of a mesenchymal phenotype. Interestingly, while most colorectal tumors exhibit increased intracellular β-catenin levels, not all show mesenchymal characteristics [36,37].

**Figure 3:**
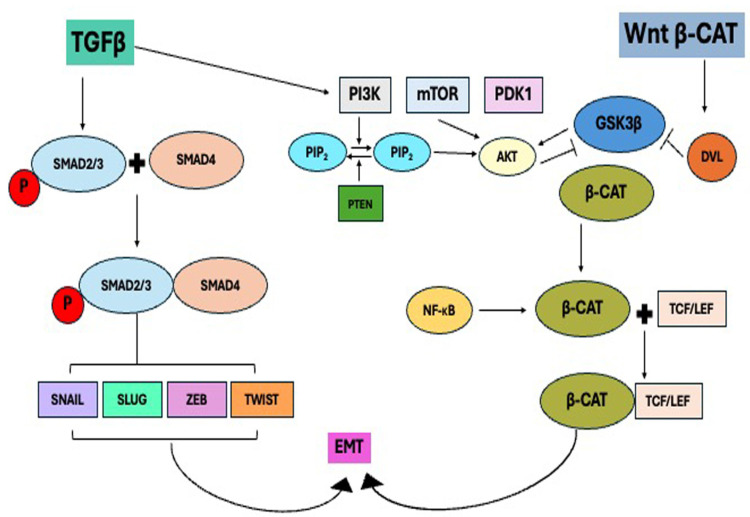
EMT regulation by TGFβ and Wnt signaling pathways. Smad proteins are activated by TGFβ activates to promote transcription of EMT transcription factors such as Snail and Slug. Furthermore, the PI3K/AKT pathway facilitates TGFβ crosstalk with the Wnt pathway. Wnt signaling maintains the expression of β-catenin, which enables it to combine with TCF/LEF to form a transcriptional complex that increases the expression of EMT regulators like Snail, Slug, and vimentin [34].

β-catenin integrates various signaling pathways to drive cancer metastasis and developmental processes by coordinating cytoskeletal remodeling, cell mobility, and invasiveness by connecting Wnt activation to EMT programs. This includes the Transformation Growth Factor β (TGFβ) signaling pathway. EMT can be repressed if these pathways and their associated components are inhibited (Figure 3) [11,34,36]. Therefore, β-catenin may function as a molecular node that facilitates communication between Wnt and additional EMT signaling pathways [38,39,40]. 

## 5. The TGF-β Signaling Pathway

TGF-β (transforming growth factor-beta) is a cytokine known to regulate several cellular mechanisms. TGFβ is inhibitory for cell proliferation and growth under normal circumstances. These cytokines are known for regulating inhibition of cell proliferation and promoting tissue fibrosis via production of extracellular matrix.

TGF-β plays an important dual context-dependent role in cancer. In less advanced cancers, TGF-β inhibits cell proliferation, leading to tumor suppression. This growth-suppressive effect is rendered through regulation of multiple processes such as immune response and cell growth.

However, in advanced cancers, the suppressive effect of TGFβ is lost, and the TGFβ pathway promotes tumor growth and progression to an aggressive phenotype. In this context, TGF-β promotes EMT, leading to matrix deposition and immune response modulation. EMT is driven by TGF-β and several other signaling molecules, including Wnt, playing and important role in the invasion and metastasis of cancer [41,42]. TGFβ is also used by tumor cells as a protective shield against the immune response of the body, which effectively aims to destroy the tumor cells. This subsequently leads to enhanced growth and progression of these cells [42,43]. 

TGF-β activates Smad and non-Smad pathways (PI3K/AKT) via TbRII and TbRI, which are type II and type I serine–threonine kinase receptors. Canonical Wnt and TGF-β1 pathways stimulate each other through PI3K/Akt, which is an important pathway that regulates the growth, survival, and proliferation of cells, and the dysregulation of this pathway is associated with many cancers. Smad and non-Smad pathways further regulate EMT and the progression of cancer by cooperating with other signaling pathways like canonical Wnt and TGF-β1 pathways directly or indirectly [44,45]. One of the hallmarks of EMT is E cadherin, an integral part of EMT. Various transcriptional factors such as Snail1, Snail2 (Snail family transcriptional repressor 2), ZEB1 (Zinc finger E-Box binding homeobox 1), and twist (twist family BHLH transcription factor 1) are known to bind in the promoter region of E cadherin. It is mainly Snail1 that is reported to be upregulated by TGF-β through both Smad-dependent and -independent signaling. Additionally, Snail1 downregulates the expression of E cadherin and promotes the expression of mesenchymal markers, for instance vimentin, leading to the weakening of cell-to-cell adhesion [41,46,47]. 

## 6. The Metastatic Cascade

The ability of cancer cells to invade surrounding tissue, spread throughout the body, and develop new tumors (metastasis) in distant organs is what distinguishes the malignant form of the disease, and which accounts for over 90% of cancer-related fatalities. The series of events that result in metastases in distant organs are referred to as the metastatic cascade [9,48]. In epithelial cancers (carcinomas), it begins with the tumor cells losing their ability to adhere to one another, detach from other cells in the primary tumor, and migrate/invade into the surrounding tissue. Cells then invade through the surrounding extracellular matrix (ECM) to the capillary beds and intravasate into the lymphatic and blood vascular system. Cancer cells have to endure/survive the severe conditions in the bloodstream, including shear forces of the blood and immune surveillance in order to spread throughout the body. Surviving cells subsequently extravasate into the organ parenchyma at a secondary site. When they reach the “metastatic niche”, they either become individual disseminated tumor cells (DTCs) or multicellular micro-metastases, which then begin to multiply/proliferate to produce macroscopic metastases [40,43,49,50].

Metastatic cells can also enter a long-lasting latent cell state. Only 0.01% of tumor cells that reach the bloodstream are thought to have the capacity to develop into secondary cancers [40]. 

This sequence of events occurs as the carcinoma cells undergo a process of EMT to form a mesenchymal group of cells that are able to detach from neighboring cells, migrate, and invade the ECM. EMT is a multi-stage process that involves the slow modification of the architecture and functional capacities of epithelial cells. The cells go into a low proliferative state with a spindle-like form and increased motility, invasion, and survival. They also lose their apical–basal cell polarity and epithelial cell–cell connections. While the reverse process, known as MET, is thought to enhance metastatic expansion once cancer cells have reached distant organs, the aberrant activation of an EMT encourages tumor cell invasion and dispersion. It appears that the EMT/MET processes-mediated cell plasticity is a crucial aspect of both physiological and pathological processes, including cancer, tissue fibrosis, embryogenesis, and tissue homeostasis [1,40,48,51,52]. 

## 7. Role of EMT in Cancer Progression and Metastasis

**Figure 4:**
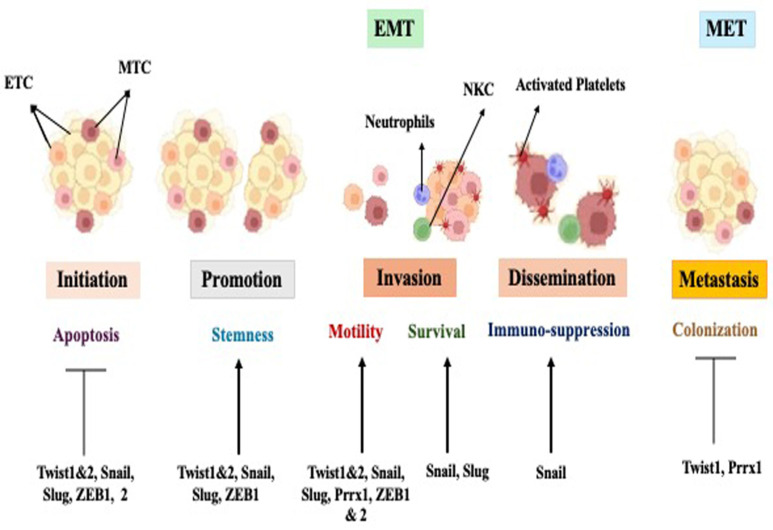
EMT in tumor progression. EMT-inducing transcription factors not only contribute to increasing mobility and migration of cells that cause cancer cell invasiveness but also contribute to multiple important functions that lead to initiation and progression of tumors. The transcription factors inducing EMT abrogate oncogene-mediated senescence and apoptosis during the initial stage of tumorigenesis. These transcription factors also confer stemness in tumor cells and facilitate immunosuppression and cell survival. In contrast, EMT suppression is required in colonization or clonal outgrowth at metastatic sites by down regulation of EMT-inducing transcription factors such as Twist1 and Prrx1 (paired related homeobox 1). Hence, EMT supports the invasive phenotype/phase of cancer metastasis, while MET supports the colonization and formation of metastases in a distant organ [20].

What is the role of EMT in tumor progression? Is EMT required in metastasis? The development of metastases involves primary cancer cell local invasion to the surrounding environment, intravasation into the blood vascular system via capillary networks, dissemination through the bloodstream, extravasation into distant organs, and colonization to form metastatic out-growth (metastases) (Figure 4) [20].

Several in vitro and in vivo studies have defined the role of EMT in cancer cell migration and invasion. These studies have shown that the morphology of cells at the tumor invasive front is frequently de-differentiated, with a corresponding increase in the expression of mesenchymal markers and a decrease in epithelial markers and cell–cell adhesion junctions. In addition, there is an increase in metastasis when EMT is induced in cancer cells: through induction of TGFβ signaling, overexpression of EMT promoting transcription factors, or the disruption/down regulation of E-cadherin function at cell adherence junctions. In contrast, decreased metastasis has been observed when EMT is inhibited: by siRNA-mediated knockdown or genetic deletion of genes that induce EMT [40,53]. It has been demonstrated that disruption/alteration of EMT-inducing genes and their respective proteins influences the process of metastasis. Additionally, circulating tumor cells (CTCs) extracted from the blood of metastatic breast cancer patients demonstrate more mesenchymal cell marker expression compared to primary tumor cells, and this expression is correlated with a poor clinical outcome [54]. In vivo lineage tracing of tumor cells supports these findings, in a Kras/p53-driven mouse model of pancreatic cancer, where circulating and invading tumor cells exhibit a distinct mesenchymal phenotype [36,55].

Recent reports suggest that while EMT is not important for the colonization and development of metastases at distant sites, EMT of primary epithelial tumor cells is mandatory for the early stages of the metastatic cascade leading up to organ extravasation. It should be pointed out that the process of MET is mandatory in the process of the colonization of distant organs. The significance of a reversible EMT/MET process for the successful growth of distant metastases is illustrated in a mouse model of squamous cell carcinoma, whereby the activation of the EMT-inducer Twist1 in the primary tumor causes tumor cells to dissociate from the primary tumor, invade the microenvironment, and intravasate into the bloodstream for dissemination. However, the key to successful colonization and formation of viable metastases involves Twist1 inactivation and the subsequent induction of an MET at the metastatic site. Additionally, MET could account for the observed differentiated phenotype of metastatic lesions, which is similar to that of primary tumors [40].

The ability of tumor cells to acquire the ability to migrate and invade the surrounding matrix has long been attributed to the epithelial–mesenchymal transition (EMT) of tumor cells, often used as a surrogate to illustrate EMT’s role in metastasis [36,56]. When epithelial cells undergo EMT, they not only lose their apical–basal polarity and break down cell–cell contacts like tight junctions (TJs), adherents’ junctions (AJs), and desmosomes, but their actin cytoskeletal structure also undergoes reorganization. This reorganization enables the cells to adopt a spindle-shaped mesenchymal morphology and enhances their motility by facilitating the formation of protrusions. Various mechanisms contribute to this process, including cytoskeletal reorganization, altered expression of cell adhesion molecules, and degradation of the basement membrane through the activation of Matrix metallopeptinase 2 (MMP-2) (Matrix metallopeptindase 2) and Matrix metallopeptinase 9 (MMP-9) (Matrix metallopeptidnase 9), as well as sustained autocrine growth factor signaling to evade apoptosis and/or anoikis [57,58,59]. Studies utilizing mouse models of breast and skin cancers have highlighted the significance of activating an EMT program for primary tumor cells to disseminate into the lungs. However, once disseminated, these cells need to subsequently reverse the EMT program and regain epithelial characteristics to efficiently form macroscopic metastases. In various carcinomas, the experimental activation of the EMT program has been shown to significantly enhance the ability of cancer cells to form filopodia-like protrusions. These protrusions facilitate the migratory and invasive behavior of EMT-activated cells, enabling them to proliferate after extravasation and, ultimately, promote the seeding of metastatic lesions [36,60].

### Models of Metastasis

Two forms/models of metastasis are proposed to integrate the new observations in this field. The first model, known as the “plasticity type I” metastasis model, postulates that tumor cells are responsive to signals from the surrounding environment and that metastases formation is driven by dynamic EMT/MET processes. The second model, dubbed the “genetic type II” metastasis model, postulates that tumor cells experience a permanent, irreversible EMT, take on characteristics of stem cells, and lose their ability to change phenotypically as a result of genetic changes. These genetic changes compel cancer cells to colonize distant organs and proliferate without requiring MET. The coexistence of mesenchymal and epithelial markers in a cancer cell characterizes the first model, known as increased EMT/MET plasticity, which is supported by the recent application of novel analytical technologies. This model is also referred to as a partial EMT or a hybrid epithelial–mesenchymal phenotype [40,61,62].

## 8. EMT and Circulating Tumor Cells (CTCs)

CTCs specifically refer to tumor cells that have detached from a primary tumor and entered the bloodstream. Carcinoma cells that have infiltrated into the bloodstream (intravasated) exhibit incomplete EMT activation and express both epithelial and mesenchymal markers. Considered as precursors of metastasis, the molecular characteristics of CTCs serve as a valuable tool to elucidate the mechanisms underlying malignant spread. Mesenchymal CTCs have been found to be more abundant in patients affected by progressive tumors [54,63]. Moreover, the presence of CTCs during primary prostate cancer has been associated with alterations in E-cadherin expression [63,64]. 

## 9. EMT and Tumor Angiogenesis

The progression of tumors and their ability to spread are significantly influenced by two key factors. The first is epithelial–mesenchymal transition (EMT), which allows tumor cells to invade and migrate and angiogenesis. The second is angiogenesis, the formation of new blood vessels, which plays a pivotal role in advancing tumor growth and facilitating metastasis by transitioning tumors from a non-vascular to a vascular phase, known as the angiogenic switch.

EMT in tumor cells can promote angiogenesis by secreting pro-angiogenic factors, such as vascular endothelial growth factor (VEGF) (vascular endothelial growth factor), fibroblast growth factors (FGFs), and MMPs. These factors can stimulate endothelial cells (the cells lining blood vessels) to proliferate and form new vessels. EMT-driven tumor cells can directly interact with the tumor vasculature, promoting angiogenesis through signaling pathways like the neurogenic locus notch homolog protein (Notch) (neurogenic locus notch homolog protein) and TGF-β pathways [65]. 

On the other hand, tumor-induced angiogenesis generates areas of hypoxia within the tumor, which in turn triggers EMT in surrounding tumor cells. Hypoxia and nutrient deprivation act as stress signals that drive the transition from an epithelial to a mesenchymal state, allowing tumor cells to escape from the primary tumor and invade new tissue. Additionally, newly formed blood vessels close to the tumor vicinity can provide both a physical route for tumor cells to enter circulation (intravasation) and a source of pro-inflammatory signals that enhance EMT.

Research has shown a correlation between the levels of vascular endothelial growth factor (VEGF) and epidermal growth factor receptor (EGFR) with changes in Twist2 expression and the reduction in E-cadherin levels.

### 9.1. Impact of EMT and Angiogenesis on Metastasis

The role of EMT and angiogenesis on metastasis are given below.

#### 9.1.1. Metastatic Colonization

Both EMT and angiogenesis are crucial for the colonization of distant tissues. EMT allows tumor cells to detach from the primary tumor and invade local vasculature, while angiogenesis provides the necessary vascular access at the primary site and the blood supply to support the growth of metastases at secondary sites.

#### 9.1.2. Resistance to Treatment

Tumors that have undergone EMT often become more resistant to chemotherapy and targeted therapies. Additionally, the pro-angiogenic environment created by tumor vasculature may contribute to resistance by providing an influx of immune cells and growth factors that protect tumor cells from treatment-induced damage.

#### 9.1.3. Endothelial–Mesenchymal Transition (EndMT)

There is also a phenomenon known as endothelial–mesenchymal transition (EndMT), where endothelial cells, which line blood vessels, undergo a transformation similar to EMT. EndMT contributes to angiogenesis and can play a role in metastasis by enhancing the ability of endothelial cells to promote tumor cell invasion and migration.

Moreover, the activation of VEGF receptor-1 (VEGFR-1) has been linked to EMT, enhancing tumor cell motility and invasiveness. This is particularly evident in human pancreatic carcinoma. Studies utilizing xenografts of pre-invasive cells have further elucidated the intricate mechanisms underlying the relationship between EMT and tumor angiogenesis.

## 10. EMT and Inflammation

The acquisition of EMT-like traits in cancer cells can be facilitated by various inflammatory mediators that include soluble factors, oxidative stress, or hypoxia. Studies have shown a correlation between the presence of tumor-associated macrophages and EMT-like features in various cancers including gastric cancer, non-small cell lung cancer (NSCLC), and head and neck cancer [66]. 

In hepatocellular carcinoma, co-culture experiments have demonstrated that macrophages induce EMT in cancer cells, either through an Interlukin 8 (IL-8) -dependent mechanism or a TGF-β-dependent mechanism. Additionally, tumor necrosis factor alpha (TNF-α), in conjunction with TGF-β or other inflammatory factors, has been found to induce EMT [67,68]. 

A reciprocal relationship between IL-8 and EMT has been established, forming a mutual loop where IL-8 and EMT programs sustain each other within the tumor microenvironment. Furthermore, EMT associated with inflammation has been linked to advanced stages of cancer progression. In patients with inflammatory breast cancer, there is a correlation between immune activation and the presence of circulating tumor cells exhibiting EMT characteristics [67,68]. 

## 11. Regulation and Modulation of EMT

Several factors that influence EMT regulation and modulation are described below.

### 11.1. Micro-Environmental Cues

#### 11.1.1. Hypoxia as a Key EMT Regulator

Hypoxia, or low oxygen levels in the tumor microenvironment, plays a critical role in driving epithelial–mesenchymal transition (EMT), a key process in cancer progression. Under hypoxic conditions, the hypoxia-inducible factor-1 alpha (HIF-1α) is stabilized, triggering the activation of EMT-inducing transcription factors like Snail, Twist, and ZEB1. These factors drive the transition of cancer cells from an epithelial to a mesenchymal state, which enables them to detach from the primary tumor and enhances their motility and invasiveness, to spread to distant sites [69,70]. 

The implications of hypoxia-induced EMT are profound. In cancers like oral squamous cell carcinoma (OSCC), hypoxia promotes a more aggressive phenotype, facilitating tumor invasion and metastasis [69]. Moreover, hypoxia-induced EMT also confers stem-cell-like properties on cancer cells, further enhancing their ability to survive and proliferate in adverse conditions [71,72]. 

#### 11.1.2. Extracellular Matrix as a Regulator

The extracellular matrix (ECM), a complex network of proteins and polysaccharides, undergoes significant remodeling in tumors, which affects the tumor’s density, stiffness, and composition, all of which play pivotal roles in regulating EMT. Increased ECM density weakens cell–cell adhesions by disrupting adherens junction proteins such as E-cadherin, promoting cell–cell detachment in the transition from epithelial to mesenchymal phenotypes [73,74]. Moreover, increased ECM stiffness activates mechanotransduction pathways, including integrin and focal adhesion kinase signaling, which facilitate EMT [74,75]. Stiffer ECMs promote cellular migration and invasion while activating EMT transcription factors such as Snail, Twist, and ZEB1, leading to a loss of epithelial characteristics. Additionally, the ECM undergoes constant remodeling, primarily through enzymes like matrix metalloproteinases (MMPs), which degrade ECM components and enable cancer cell invasion. ECM remodeling in tumors is characterized by increased collagen synthesis and deposition, usually accompanied by the increased cellular levels of remodeling enzymes and other components such as matrix metalloproteinases (MMPs), urokinase plasminogen activator (UPA), lysyl oxidase (LOX), lysyl oxidase-like proteins (LOXLs), WNT1-inducible signaling pathway proteins (WISPs), and others. These enzymes can catalyze specific ECM components as substrates to control tissue stiffness and cell–matrix interactions through their unique biochemical and physical properties. These enzymes are essential for ECM remodeling. In order to facilitate tissue remodeling, cell migration, and cancer invasion, UPA, a serine protease, transforms plasminogen into plasmin, which is a protease that breaks down fibrin and other ECM components and promotes the activation of additional matrix-degrading enzymes. LOX and LOXLs cause catalyzation of the cross-linking of elastin fibers and collagen, which stabilizes and stiffens the extracellular matrix. LOX and LOXLs support the structural integrity of the ECM, whereas UPA encourages ECM remodeling and degradation. By striking a balance between stiffening and breakdown, these enzymes work together to modify the ECM in pathological settings such as cancer, facilitating tumor invasion, metastasis, and progression [76]. 

### 11.2. Cellular Interactions

#### 11.2.1. Cell–Cell Interactions

Cell–cell interactions play a crucial role in regulating EMT, significantly influencing the tumor microenvironment and the behavior of cancer cells. Kamińska et al. emphasized that interactions between cancer cells and their neighboring cells, including stromal and immune cells, are fundamental to tumor behavior [77,78]. These interactions can either promote or inhibit EMT, depending on the cellular context and the signals exchanged. Key mechanisms facilitating these interactions include adhesion molecules like cadherins and integrins, which are essential for maintaining tissue architecture and regulating critical signaling pathways. While Kamińska et al. suggest that these adhesion proteins influence cell proliferation and survival, Arias et al. highlight the role of signaling pathways such as TGF-β, Wnt, and Notch in mediating communication between epithelial and mesenchymal cells [44,77,79]. The tumor microenvironment is significantly shaped by cell–cell interactions; cancer cells can modify their surroundings, affecting the behavior of stromal cells and immune cells, which in turn supports tumor growth and metastasis. This dynamic interplay fosters a microenvironment conducive to EMT [77,78]. 

#### 11.2.2. Paracrine Signaling

Paracrine signaling, a form of cellular communication where signaling molecules released by a cell influence neighboring cells, plays a crucial role in regulating EMT. Paracrine factors, such as cytokines and growth factors, modify the behavior of adjacent cells. Gonzalez and Medici emphasize that pathways like TGF-β and Wnt are frequently activated in a paracrine manner to promote EMT in surrounding epithelial cells [11]. For instance, TGF-β released by stromal cells can induce EMT in nearby cancer cells, increasing their invasive potential. The interaction of key signaling pathways is critical in this context: Thomson et al. highlight the importance of crosstalk between various signaling pathways, noting that paracrine signals can integrate multiple inputs to regulate EMT [80,81]. The cooperation between TGF-β and Wnt signaling exemplifies this, as Wnt can enhance the effects of TGF-β, further promoting mesenchymal characteristics in epithelial cells. The interactions between cancer cells and surrounding stromal cells facilitate the release of paracrine factors that support EMT, underscoring the role of the microenvironment in shaping the signaling landscape that drives EMT in cancer progression [82]. 

## 12. Therapeutic Strategies Targeting EMT

Therapeutic strategies targeting EMT in cancer focus on disrupting the molecular pathways that facilitate the transformation of epithelial cells into mesenchymal cells, a critical step in cancer metastasis and drug resistance. One key approach is targeting EMT-related transcription factors, such as Snail, Slug, and Twist, which regulate EMT by suppressing epithelial markers and promoting mesenchymal phenotypes. Specifically, inhibitors targeting Snail, Slug, and Twist have shown promising results in reducing cancer cell migration, invasion, and drug resistance. Inhibition of Snail, for example, has been shown to block EMT and tumorigenesis, underscoring the potential of these molecules as therapeutic targets [21,60,83]. 

Another key strategy involves blocking EMT-related signaling pathways, particularly the TGF-β pathway, which is a major driver of EMT in various cancers. TGF-β inhibitors, such as SB-431542, have been shown to effectively suppress EMT and reduce tumor progression in preclinical studies [41,47,84]. Additionally, tyrosine kinase inhibitors (TKIs), which target receptor tyrosine kinases involved in EMT signaling (such as Wnt β-catenin, TGF-β signaling and notch signaling), have demonstrated efficacy in cancer therapy by inhibiting growth factor-mediated EMT signaling [85,86,87]. TKIs have become an essential component of cancer treatment strategies, particularly in targeting the aberrant signaling that drives EMT.

Epigenetic modulation also offers a novel avenue for targeting EMT, as epigenetic changes, such as histone acetylation and DNA methylation, play a crucial role in regulating EMT-related gene expression. Histone deacetylase (HDAC) inhibitors, for example, can reverse the epigenetic silencing of epithelial genes, thereby inhibiting EMT [88,89]. Similarly, DNA methyltransferase inhibitors (DNMTis) have been explored for their ability to modulate EMT by reversing abnormal DNA methylation patterns in cancer cells, leading to the re-expression of tumor suppressor genes and the inhibition of metastatic traits [90,91]. These epigenetic therapies offer a promising approach to reprogramming cancer cells and suppressing EMT-driven metastasis.

Overall, therapeutic strategies targeting EMT involve a multifaceted approach, ranging from direct inhibition of transcription factors to blocking key signaling pathways and utilizing epigenetic modulators. These approaches aim to interrupt the EMT process, reduce metastasis, and improve the efficacy of cancer treatments.

### 12.1. CRISPR/Cas9-Mediated Gene Editing

In recent years, the CRISPR-Cas system has significantly advanced our ability to edit genomes, helping us to identify and validate essential genes involved in signaling pathways related to EMT. Different approaches including gene knockout, knockdown, knock-in, and epigenetic regulation are achieved by the CRISPR/Cas technology to study EMT-related genes and pathways and identify novel drugs to control the EMT process [92].

### 12.2. miRNA-Based Therapies

RNA interference or RNAi is a powerful molecular tool that is used extensively to study, in vitro, the role of genes involved during EMT. However, in vitro identification of EMT-promoting genes has proven to be impractical due to its non-specificity to the target cell type. Recently, microRNAs (miRNAs) were shown to regulate either a single step or multiple steps of metastasis by downregulating the expression of their target genes. miRNAs bind to either perfect or imperfect complementary sequences in target mRNAs at the miRNA recognition elements (MREs) located in their 3′UTR via a “seed” region, resulting in the cleavage of target mRNAs or inhibition of their translation. Notably, RNAs that compete for the same MREs, known as competing endogenous RNAs (ceRNAs), are thought to play a role in regulating important oncogenes and tumor suppressor genes. These ceRNAs can be mRNAs or RNAs derived from pseudogenes and other non-coding genes. For example, RNAs that share miRNA binding sites with PTEN, like its pseudogene PTENP1, enhance PTEN expression by acting as endogenous miRNA decoys or sponges. Recently, miR-205 and the miR-200 family (including miR-200a, miR-200b, miR-200c, miR-141, and miR-429, which share a common seed sequence) were identified as new epithelial markers and inhibitors of epithelial-to-mesenchymal transition (EMT) and stem cell properties. Members of the miR-200 family promote mesenchymal-to-epithelial transition (MET) and inhibit EMT by directly targeting the mRNAs of ZEB1 and ZEB2. In contrast, ZEB1 represses the transcription of miR-200 genes by binding directly to their promoter region, creating a double-negative feedback loop. The expression of the miR-200 family is lost in areas of metaplastic breast cancers that lack E-cadherin, while ZEB1 and ZEB2 are highly expressed in invasive mesenchymal cells [3,93]. 

### 12.3. Exosome-Based Strategies

In addition to the interactions among various intracellular signaling pathways that regulate EMT, extracellular modulators in the tumor microenvironment also drive tumor cells to undergo this process. Extracellular vesicles (EVs) have gained attention as important inducers of EMT. These vesicles, which include exosomes and microvesicles, transport proteins, nucleic acids, lipids, and other small molecules that can stimulate EMT in recipient cells. Exosomes, in particular, have been the focus of numerous studies due to their significant role in facilitating intercellular communication. By promoting the exchange of molecular signals between cells, exosome-based strategies control EMT and influence the EMT process. In EMT, signaling molecules including TGF-β, miRNAs, mRNA, and proteins like Snail, Slug, and Twist found in exosomes from a variety of sources, including tumor cells, fibroblasts, or endothelial cells can activate EMT-related pathways in target cells. For instance, by delivering TGF-β or miRNAs that stimulate mesenchymal gene expression, exosomes generated from cancer cells can cause EMT in healthy epithelial cells, increasing cell motility, invasion, and metastasis. Exosomes are important participants in the development and dissemination of cancer because they can alter the microenvironment of tumor by improving ECM remodeling, encouraging angiogenesis, and increasing cell survival during the EMT process [94]. 

### 12.4. Immunotherapy

Immunotherapy is a treatment that uses the patient’s own immune system to fight cancer. Molecular characterization of non-responsive cancers indicates that an embryonic program called EMT, typically dormant in adults, can be activated under certain selective pressures. This activation may contribute to the resistance of these cancers to chemotherapy and immunotherapy. Additionally, EMT can promote tumor metastasis, which suppresses the activity of cytotoxic T cells that infiltrate the tumor, ultimately leading to the failure of immunotherapy. EMT broadly upregulates various immune checkpoint and inflammatory molecules, leading to CD8+ T cell exhaustion. This highlights several potential mechanisms behind therapeutic resistance to immunotherapy in mesenchymal tumors. EMT is a complex process with multiple dimensions, and these factors can be exploited by malignancies in response to selective pressures such as cytotoxic therapies, biological treatments, hypoxia, metabolic changes, and immune surveillance. Consequently, there is an urgent clinical need for targeted, biomarker-directed therapies to tackle both primary and acquired resistance to immunotherapy. Incorporating targeted agents and validated biomarkers that modulate immune suppression could widen the patient population responsive to immune checkpoint inhibitors and help overcome immunotherapy resistance [95]. 

## 13. Conclusions

EMT supports the formation of new organs and the regeneration of damaged tissue. The dynamic nature of cellular EMT is crucial for both wound healing and embryogenesis. EMT is extremely important for the growth, invasion, and metastasis of tumors. Several EMT transfection factors including Snail 1, Snail 2 (Slug), Twist1, Twist2, ZEB1, ZEB2, PRRX1, GOOSECOID (GSC), E47 (helix loop transcription factor), FOXC2 (Forkhead X box protein P2), SOX4, (SRY box transcription factor 4), SOX9 (SRY box transcription factor 9), HAND1 (heart and neural crest derivatives expressed 1), and HAND2 (heart and neural crest derivatives expressed 2), promote mesenchymal properties in epithelial tumor cells. These transcription factors induce signaling pathways, such as the canonical Wnt β- catenin pathway and TGFβ signaling pathways, that promote cancer growth, progression, and invasion. EMT also plays vital role in establishing an immunosuppressive microenvironment for tumor cells. In addition, EMT induces resistance towards immunotherapy, which effectively enhances the ability of advanced cancer cells to invade their microenvironment for successful dissemination.

Despite developments in revealing the molecular mechanism of EMT and its involvement in cancer progression, scientists struggle to convert these results into practical treatments. EMT-focused therapies exhibit substantial therapeutic potential, while difficulties and challenges such as the heterogeneity of tumors, the reversible nature of EMT, and resistance to treatments also remain essential areas for exploration. However, developing a better understanding of the specific molecular pathways and biomarkers in EMT would be invaluable as could inform the design of personalized and targeted therapeutic treatments.

Further studies are needed to establish a better understanding of the biological significance of EMT in different types of cancer as well as at different phases of cancer progression. This will enable the design of targeted therapies against regulators of EMT, which could inform the design of clinically feasible therapeutic strategies that specifically target tumor cells undergoing EMT.

Researchers should focus on identifying developing EMT biomarkers that may be used for diagnosis of disease to act as indicators of advancement; of the disease’s response to therapies; and in the investigation, design, and implementation of combination of therapies that could target EMT and important pathways associated with disease progression in cancer. Additionally, understanding interaction/s between the tumor microenvironment and EMT may lead to the discovery of advanced therapies. If these challenges are addressed, there would be opportunities to create EMT-targeted therapies in laboratory settings that could then be introduced in clinical practice and to improve patient outcomes for patients in with severe metastatic cancers with advanced disease progression.

## Figures and Tables

**Table 1:** Summary of EMT markers.

Increased Proteins	Decreased Proteins
N-cadherin	E-cadherin
Vimentin	Desmoplakin
N-cadherin	Occludin
MMP-9	Cytokeratin
MMP-3	
MMP-2	
Fibronectin	
Snail 1 (Snail	
Snail 2 (Slug)	
Twist	
FOX C2	
SOX 10	

**Table 2:** Expression of transcription factors in various types of cancers.

Type of Cancer	Invasiveness/Metastasis	References
Lung	Slug, ZEB 1, Twist 1	[20,22,23]
Breast	Snail, Slug, ZEB 1, ZEB 2, Twist 1, Twist 2	[20,22,24,25,26]
Head and neck	Snail, Twist 2	[27,28]
Gastric	Snail, ZEB 1, Twist 1	[23,29,30]
Prostrate	ZEB 1, Twist 1	[31,32]
Ovarian	ZEB 2	[33]
Colorectal	Snail, Slug, ZEB 1, ZEB 2, Twist 1	[34]
